# GRIK2 has a role in the maintenance of urothelial carcinoma stem-like cells, and its expression is associated with poorer prognosis

**DOI:** 10.18632/oncotarget.16259

**Published:** 2017-03-16

**Authors:** Ryuta Inoue, Yoshihiko Hirohashi, Hiroshi Kitamura, Sachiyo Nishida, Aiko Murai, Akari Takaya, Eri Yamamoto, Masahiro Matsuki, Toshiaki Tanaka, Terufumi Kubo, Munehide Nakatsugawa, Takayuki Kanaseki, Tomohide Tsukahara, Noriyuki Sato, Naoya Masumori, Toshihiko Torigoe

**Affiliations:** ^1^ Department of Pathology, Sapporo Medical University School of Medicine, Chuo-Ku, Sapporo 060-8556, Japan; ^2^ Department of Urology, Sapporo Medical University School of Medicine, Chuo-Ku, Sapporo 060-8556, Japan; ^3^ Department of Urology, Graduate School of Medicine and Pharmaceutical Science for Research, University of Toyama, Toyama-Shi 930-0194, Japan

**Keywords:** cancer stem-like cells, ALDH1, GRIK2, urothelial carcinoma

## Abstract

Cancer stem-like cells (CSCs)/cancer-initiating cells (CICs) are small sub-population of cancer cells that are endowed with higher tumor-initiating ability, self-renewal ability and differentiation ability. CSCs/CICs could be isolated as high aldehyde dehydrogenase 1 activity cells (ALDH1^high^) from various cancer samples. In this study, we isolated urothelial carcinoma CSCs/CICs as ALDH^high^ cells and investigated the molecular aspects. ALDH1^high^ cells showed greater sphere-forming ability and higher tumor-initiating ability in immune-deficient mice than those of ALDH1^low^ cells, indicating that CSCs/CICs were enriched in ALDH1^high^ cells. cDNA microarray analysis revealed that an ionotropic glutamate receptor glutamate receptor, ionotropic, kainate 2 (GRIK2) was expressed in ALDH1^high^ cells at a higher level than that in ALDH1^low^ cells. GRIK2 gene knockdown by siRNAs decreased the sphere-forming ability and invasion ability, whereas GRIK2 overexpression increased the sphere-forming ability, invasion ability and tumorigenicity, indicating that GRIK2 has a role in the maintenance of CSCs/CICs. Immunohistochemical staining revealed that higher levels of GRIK2 and ALDH1 expression were related to poorer prognosis in urinary tract carcinoma cases. The findings indicate that GRIK2 has a role in the maintenance of urothelial CSCs/CICs and that GRIK2 and ALDH1 can be prognosis prediction markers for urinary tract carcinomas.

## INTRODUCTION

Urothelial carcinomas including bladder cancer, urethral cancer, renal pelvic cancer and ureteral cancer are common urological cancers. Metastatic urothelial carcinoma (UC) is relatively sensitive to chemotherapy with a response rate of 50–70%; however, the three-year survival rate is less than 20% due to the high recurrence rate [1]. Thus, overcoming the problems of recurrence and treatment resistance is essential to cure urothelial cancer. Recent studies have revealed that cancer stem-like cells (CSCs)/cancer-initiating cells (CICs) are the major mechanisms of cancer recurrence and distant metastasis after treatments [2, 3]. Tumors are comprised of heterogeneous cell populations, and a small distinct sub-population has higher tumor-initiating ability, self-renewal ability and differentiation ability according to the cancer stem cell theory [3]. CSCs/CICs could be identified from urothelial carcinoma samples using several methods including the use of cell surface marker CD44^+^ or 67-kDa laminin receptor, side population analysis using Hoechst 33342 or Dye-cycle violet, and ALDH1 activity based on the ALDEFLUOR assay [4–8].

ALDH1 enzymes have roles in epithelial development and homeostasis. Deregulation of this class of enzymes is implicated in multiple cancers. ALDH1 is a cytoplasmic isoform of ALDH, and high levels of its activity are seen not only in hematopoietic stem cells but also in solid cancers [9–15]. Furthermore, ALDH1 enzymes are related to drug resistance, cell proliferation, cell differentiation and response to oxidative stress. Glutamate receptor, ionotropic, kainate 2 (GRIK2) belongs to an ionotropic glutamate receptor and is broadly expressed in central nerve system and plays a major role in nerve excitation [16]. GRIK2 is expressed in some normal organs including stomach, and a recent study revealed that GRIK2 transcription was repressed by hypermethylation of promoter region in gastric cancers, suggesting that GRIK2 might be a novel tumor suppressor gene in gastric cancers [17, 18]. Recently, polymorohism of GRIK2 TT (rs1335022) was associated with high risk of oral cancer in tobacco habitués, indicating that GRIK2 might be related to carcinogenesis [19]. However, there is no report describing the relation of CSC/CIC and GRIK2.

Urothelial carcinoma contains a small population of CSCs/CICs, and we previously reported that ALDH1-positive upper urinary tract carcinoma had a poor prognosis compared with that of ALDH1-negative tumors. [15] In this study, we isolated urothelial carcinoma CSCs/CICs, screened the CSC/CIC-specific genes and identified GRIK2 is preferentially expressed in urothelial CSCs/CICs. We furthermore analyzed the functions of GRIK2 by gene knockdown using siRNAs and gene overexpression, and analyzed the clinical significance of GRIK2 protein expression in urothelial carcinomas.

## RESULTS

### Isolation of UC stem-like cells based on aldehyde dehydrogenase 1A1 activity assay

**Figure 1:**
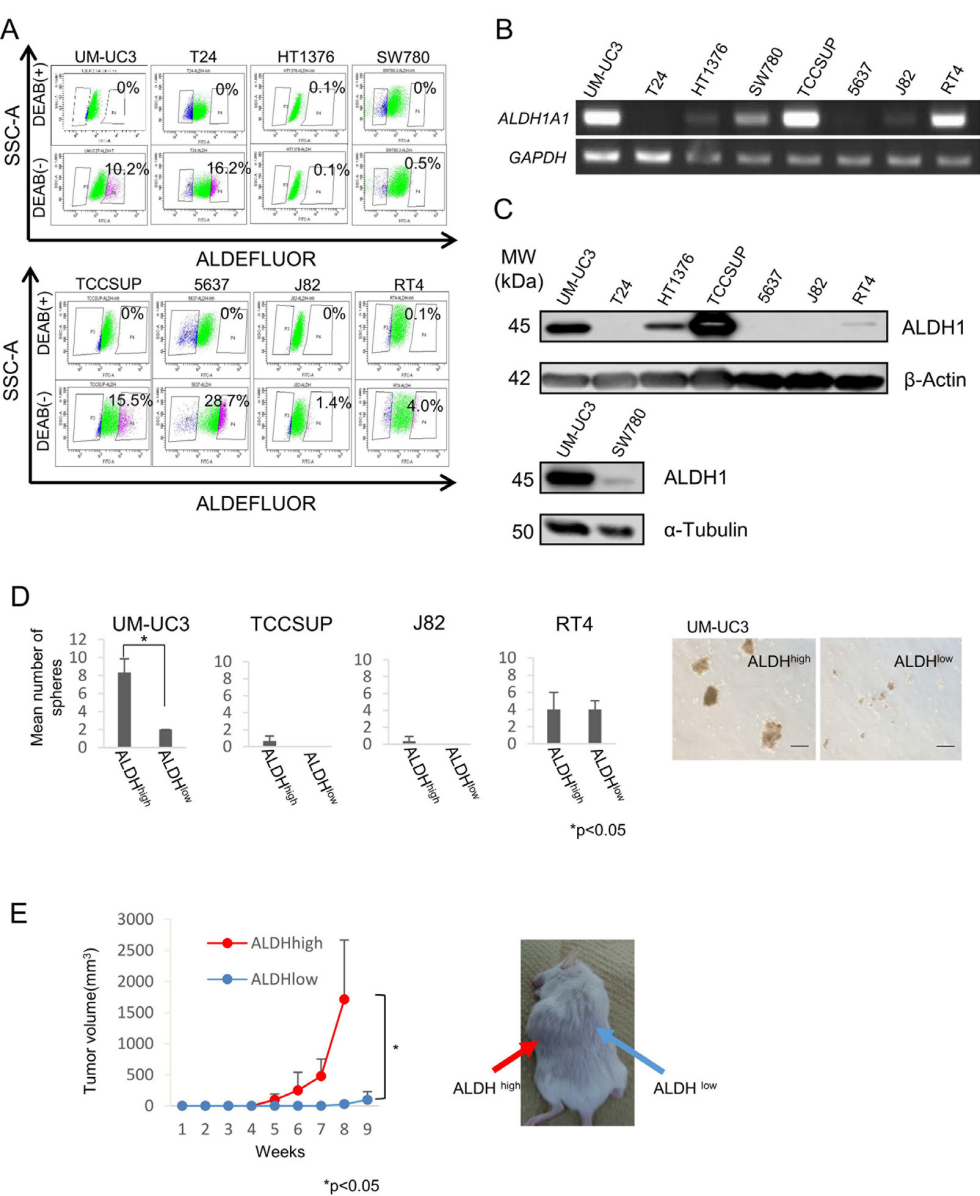
Expression of ALDH1A1 and isolation of UC CSCs/CICs (**A**) ALDEFLUOR assay of urothelial carcinoma cell lines. *SSC-A*, single strand analysis. *FITC-A*, fluorescein isothiocyanate analysis. Percent indicates ALDH1-positive rate. (**B**) RT-PCR analysis of urothelial carcinoma cell lines. Expression of *ALDH1A1* mRNA was examined by RT-PCR. *GAPDH* was used as a positive control. (**C**) Western blot analysis of ALDH1 protein. Urothelial carcinoma cell lines were analyzed with anti-ALDH1 mAb (clone: 44/ALDH). β-Actin and α-Tubulin were used as positive controls. (**D**) Sphere-forming assay. ALDH1^high^ and ALDH1^low^ cells derived from UM-UC3, TCCSUP, J82 and RT4 cells were incubated in serum-free Dulbecco's modified Eagles medium (DMEM)/F12 media with growth factors. Each value is the mean number of spheres ± SD. *P values. Black bar is 100 μm. (**E**) Tumor growth curves of ALDH1^high^ and ALDH1^low^ cells derived from UM-UC3 cells injected in NOD/SCID mice, and representative views of mouse tumors. Each value is the mean tumor volume ± SD. *P values.

Aldehyde dehydrogenase 1 (ALDH1) activity is a cancer stem-like cells(CSCs)/cancer-initiating cells(CICs) marker of various cancers [9–13]. We thus investigated whether ALDH1 activity is applicable to isolate urothelial CSCs/CICs. ALDH1^high^ cells were detectable in all urothelial carcinoma cell lines except for HT1376 and SW780 cell lines. The ratios of ALDH1^high^ cells in UM-UC3, T24, TCCSUP, 5637, J82 and RT4 cell lines were 10.2%, 16.2%, 15.5%, 28.7%, 1.4% and 4.0%, respectively (Figure 1A). However, RT-PCR and Western blot analysis revealed that *ALDH1A1* mRNA and ALDH1 protein were not expressed in T24 and 5637 cells (Figure 1B and 1C). We therefore further analyzed ALDH1^high^ cells derived from UM-UC3, TCCSUP, J82 and RT4 cells.

A sphere-forming assay was performed to analyze ALDH1^high^ cells derived from UM-UC3, TCCSUP, J82, and RT4 cells. Sphere-forming ability was evaluated using 2 × 10^2^ of sorted ALDH1^high^ and ALDH1^low^ cells in Ultra-Low Attachment Surface culture dishes. Only ALDH1^high^ cells derived from UM-UC3 cells showed higher sphere-forming ability than that of ALDH1^low^ cells (Figure 1D). To exclude mechanical damage by cell sorting, we examined sphere-forming ability in medium containing 10% FBS. Little sphere formation was observed in ALDH1^high^ cells derived from TCCSUP and J82 cells (Supplementary Figure 1). Mechanical damage caused by the cell sorter may induce cell death of TCCSUP and J82 cells.

To examine the tumorigenic potential of ALDH1^high^ cells derived from UM-UC3 cells *in vivo*, we performed xenograft transplantation of ALDH1^high^ and ALDH1^low^ cells into NOD/SCID mice. ALDH1^high^ cells initiated the formation of tumors in all of the 6 mice, whereas ALDH1^low^ cells initiated the formation of tumors in only 3 of the 6 mice. Tumors derived from ALDH1^high^ cells were significantly larger than those derived from ALDH1^low^ cells (*P* < 0.05) (Figure 1E). These results indicated that ALDH1^high^ cells derived from UM-UC3 cells were enriched with CSCs/CICs, and we therefore used UM-UC3 cells in the following analysis.

### ALDH1^high^ cells have higher invasion ability and are resistant to cisplatin

**Figure 2:**
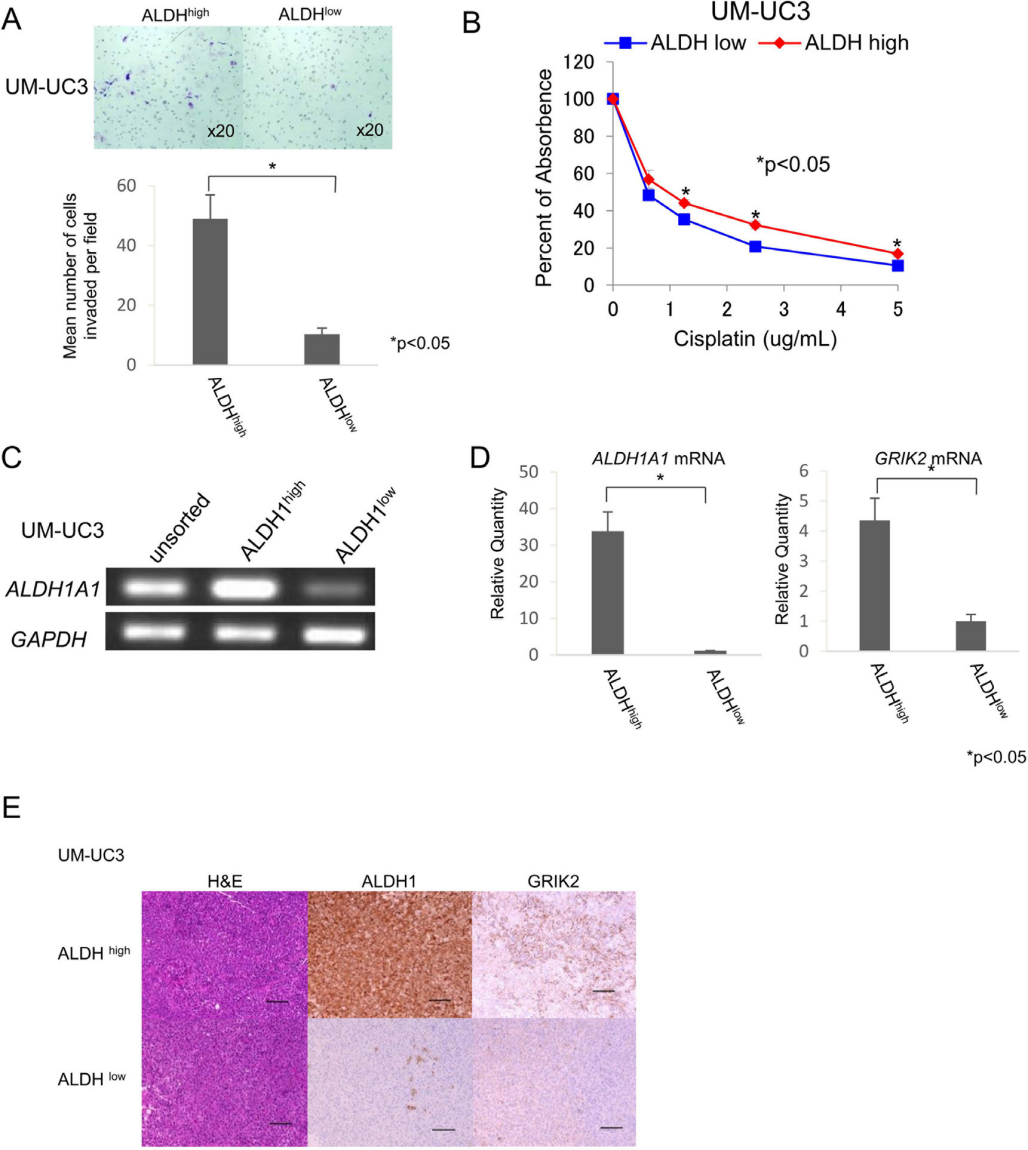
ALDH1^high^ cells have properties of CSCs/CICs (**A**) Matrigel invasion assay. Matrigel-invading cells derived from ALDH^high^ and ALDH^low^ cells of UM-UC3 cells. Magnification of images: x20. Each value is the mean number of invading cells ± SD. *P values. (**B**) Cell viability in cisplatin. Cell viability was analyzed by the WST-1 assay. Percent absorbance of ALDH^high^ and ALDH^low^ cells of UM-UC3 cells treated with cisplatin. Each value is the mean percent of absorbance ± SD. *P values. (**C**) RT-PCR analysis with unsorted, ALDH^high^ and ALDH^low^ cells of UM-UC3 cells. Expression of *ALDH1A1* mRNA was examined by RT-PCR. *GAPDH* was used as a positive control. (**D**) Quantitative real-time PCR. Relative quantities of *ALDH1A1* and *GRIK2* mRNAs of ALDH^high^ and ALDH^low^ cells of UM-UC3 cells. Each value is the mean relative quantity ± SD. *P values. (**E**) Histological images of ALDH^high^ and ALDH^low^ tumors derived from UM-UC3 cells. Hematoxylin-eosin staining (H&E), ALDH1 immunohistochemical staining and GRIK2 immunohistochemical staining of ALDH^high^ and ALDH^low^ tumors. Black bar is 100 μm.

UC has properties of local invasion and lymph node metastasis. We therefore performed an invasion assay to address the invasion ability of UC CSCs/CICs. ALDH1^high^ cells derived from UM-UC3 cells showed significantly greater invasion ability than that of ALDH1^low^ cells (*P* < 0.05) (Figure 2A). Chemotherapy is a key treatment for metastatic advanced UCs and cisplatin is the key drug for UCs. We thus analyzed the sensitivity to chemotherapy of ALDH1^high^ cells and found that ALDH1^high^ cells were more resistant to cisplatin than were ALDH1^low^ cells (Figure 2B).

### Identification of ALDH1^high^ cell-related genes

To address the gene expression profiles of ALDH1^high^ cells, we performed cDNA microarray screening using ALDH1^high^ and ALDH1^low^ cells derived from UM-UC3 cells. Approximately 50 genes were up-regulated in ALDH1^high^ cells compared with the genes in ALDH1^low^ cells (Supplementary Table 1). We screened the expression profiles in normal adult tissues by RT-PCR using specific primers for candidate genes and found that the expression level of glutamate receptor, ionotropic, kainate 2 (*GRIK2*) was expressed at relatively low levels in normal tissues (Supplementary Figure 2). Immunohistochemical staining revealed that GRIK2 was expressed in UC cells (Supplementary Figures 3A and 3B). The expression of GRIK2 in ALDH1^high^ cells was confirmed by RT-PCR (Figure 2C), quantitative real-time PCR (qRT-PCR) (Figure 2D) and immunohistochemichal staining using tumors derived from ALDH1^high^ cells and ALDH1^low^ cells (Figure 2E).

### Functional analysis of GRIK2 by siRNA-mediated knockdown

**Figure 3:**
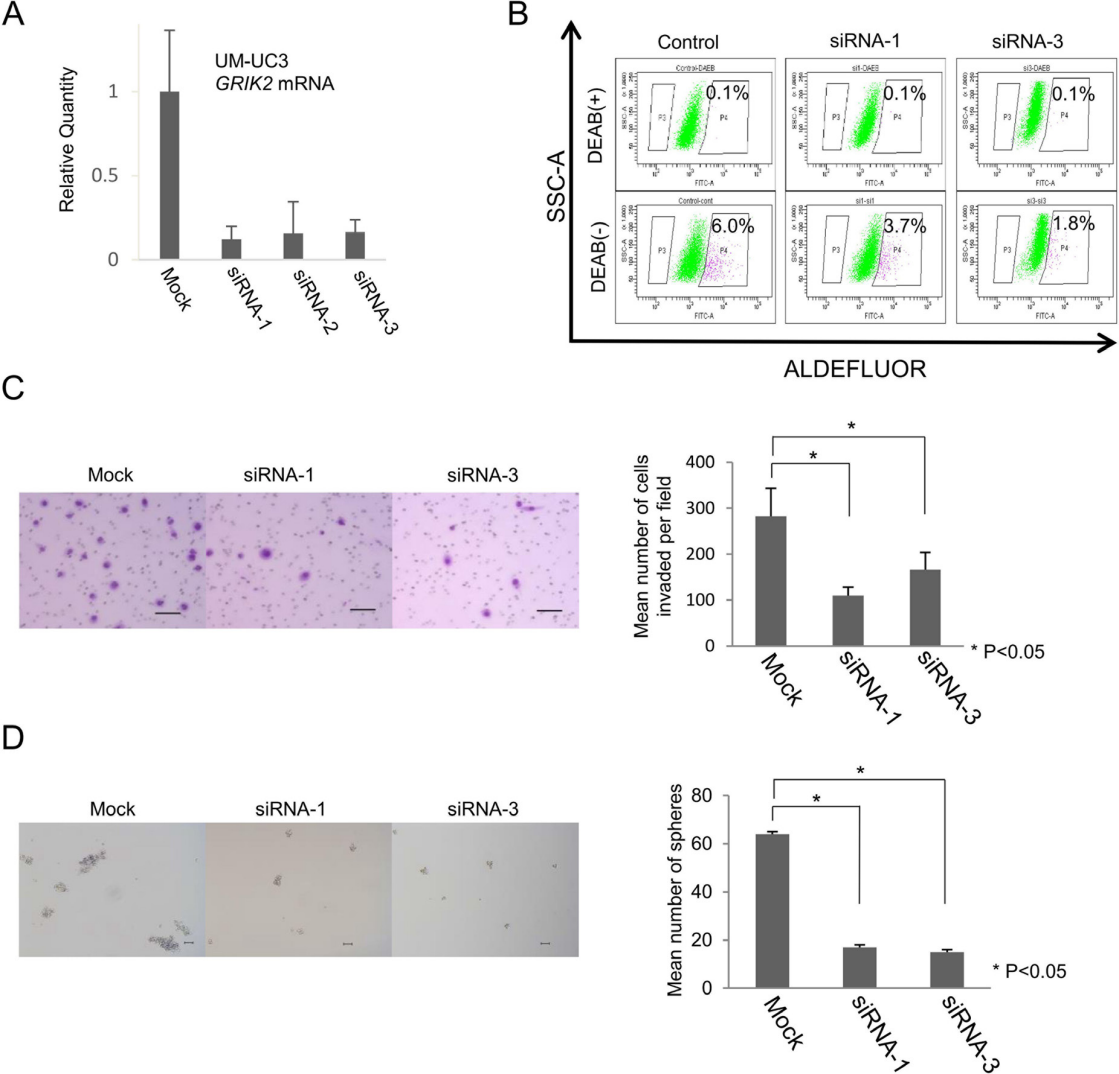
Functional analysis of *GRIK2* by siRNA-mediated mRNA knockdown (**A**) Quantitative real-time PCR. Relative quantity of *GRIK2* mRNA of UM-UC3 si-mediated mRNA knockdown cells. (**B**) ALDEFLUOR assay of UM-UC3 *GRIK2* knockdown cells. *SSC-A*, single strand analysis. *FITC-A*, fluorescein isothiocyanate analysis. Percent indicates ALDH1 positivity. (**C**) Matrigel invasion assay. Matrigel-invading cells derived from UM-UC3 *GRIK2* knockdown cells. Black bar is 100 μm. Each value is the mean number of invading cells ± SD. *P values. (**D**) Sphere-forming assay. Spheres derived from UM-UC3 *GRIK2* knockdown cells. Black bar is 100 μm. Each value is the mean number of spheres ± SD. *P values.

GRIK2 belongs to the kainate family of glutamate receptors and it is a predominant neurotransmitter receptor in the mammalian brain and is activated in various normal neurophysiologic processes. Although GRIK2 is distributed throughout the central nervous system, its physiological significance in cancer stem-like cells has not been determined yet [20]. We thus analyzed the function of GRIK2 in UC cells by *GRIK2* knockdown using siRNAs and *GRIK2* overexpression. Gene-specific knockdown of GRIK2 mRNA was confirmed by qRT-PCR (Figure 3A). To analyze the role of GRIK2 in UC CSCs/CICs, ALDEFLUOR assay, invasion assay and sphere-forming assay were performed. *GRIK2* knockdown by siRNAs decreased the ratios of ALDH1^high^ cells (Figure 3B). *GRIK2* knockdown by siRNAs significantly decreased invasion ability and sphere-forming ability (Figure 3C and 3D). A limiting dilution assay revealed that estimated CSCs/CICs frequency was significantly decreased by *GRIK2* knockdown by siRNAs (Table 1).

**Table 1:** Stem cell frequencies of GRIK2 or ALDH1A1 overexpressed and GRIK2 knocdown cells

Cells	sphere-formation/well				CSC frequency	95% CI	†*P* value
	1 cell/well	10 cells/well	100 cells/well	1000 cells/well			
UM-UC3/control siRNA	0/24	7/24	14/24	24/24	1 in 86	54.2-137	
UM-UC3/siRNA1	0/24	1/24	7/24	17/24	1 in 625	401.5-975	^*^9.18e-10
UM-UC3/siRNA2	0/24	1/24	4/24	19/24	1 in 602	386.9-937	^*^3.32e-09
UM-UC3/siRNA3	0/24	0/24	1/24	17/24	1 in 923	575.3-1481	^*^2.25e-12
UM-UC3/Mock	0/32	3/32	16/32	22/32	1 in 494	337.6-722	
UM-UC3/GRIK2	0/32	6/32	22/32	30/32	1 in 153	97.6-239	^*^3.44e-06
T24/Mock	0/32	1/32	8/32	15/32	1 in 1106	720-1698	
T24/GRIK2	0/32	1/32	12/32	21/32	1 in 642	437-943	*0.0473
T24/ALDH1A1	0/32	2/32	13/32	17/32	1 in 769	518-1141	0.188
5637/Mock	1/32	3/32	16/32	20/32	1 in 546	373-799	
5637/ALDH1A1	1/32	6/32	20/32	29/32	1 in 195	125-305	^*^2.73e-05

CSC frequency was caluculated by ELDA website. ^†^Defference with Mock cells or control siRNA transfected cells were caliculated by Chi-square test. *P < 0.05, **P < 0.01. CSC, cancer stem cell; CI, confidence interval.

### Functional analysis of GRIK2 by overexpression

**Figure 4:**
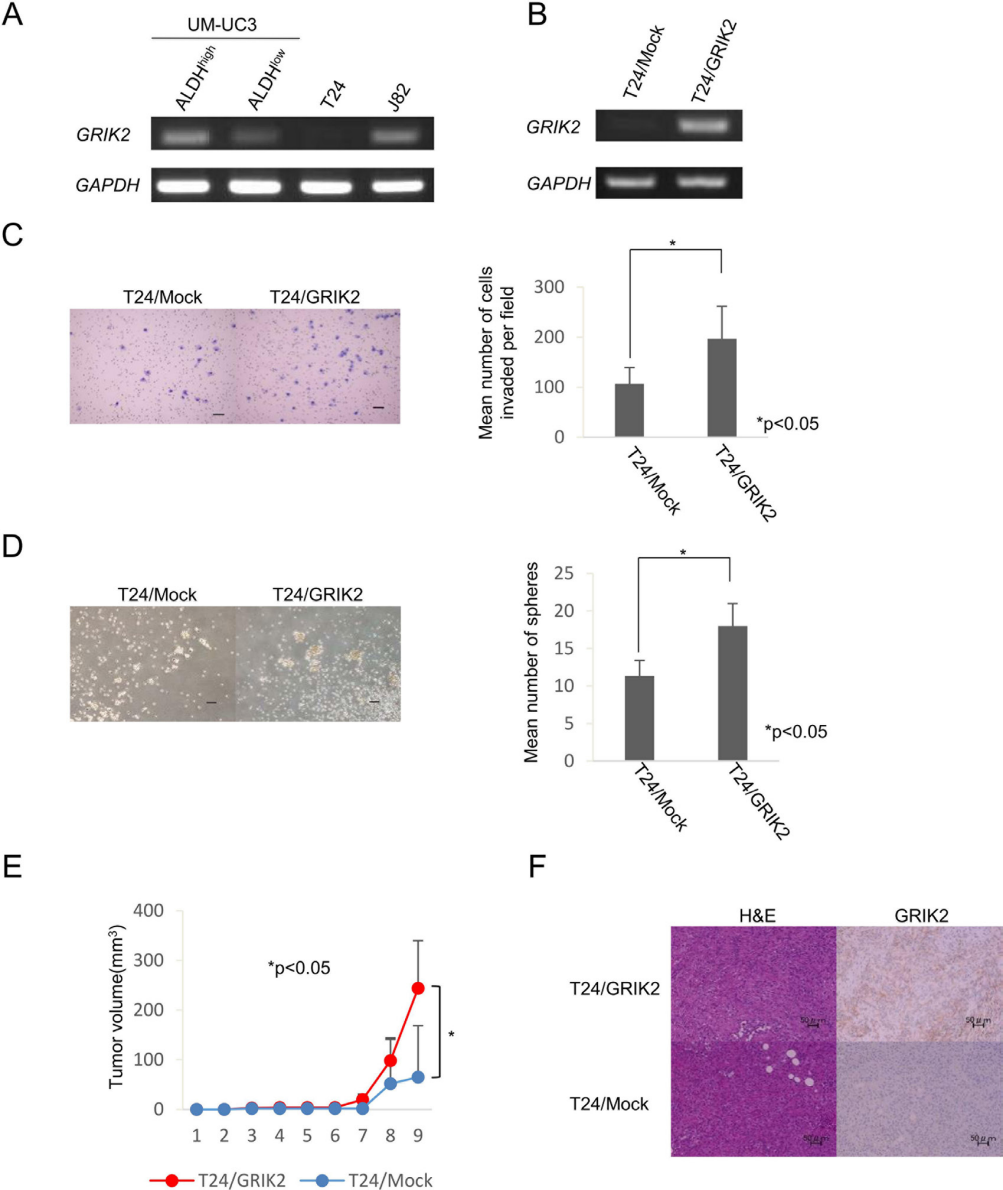
Functional analysis of GRIK2 by stable tranformants (**A**) RT-PCR analysis with ALDH^high^ and ALDH^low^ of UM-UC3, T24 and J82 cells. Expression of *GRIK2* mRNA was examined by RT-PCR. *GAPDH* was used as a positive control. (**B**) RT-PCR analysis with T24/Mock and T24/GRIK2. Expression of *GRIK2* mRNA was examined by RT-PCR. *GAPDH* was used as a positive control. (**C**) Matrigel invasion assay. Matrigel-invading cells derived from T24/Mock and T24/GRIK2 cells. Black bar is 100 μm. Each value is the mean number of invading cells ± SD. *P values. (**D**) Sphere-forming assay. Spheres derived from T24/Mock and T24/GRIK2 cells. Black bar is 100 μm. Each value is the mean number of spheres ± SD. *P values. (**E**) Tumor growth curves of T24/Mock and T24/GRIK2 cells injected in NOD/SCID mice. Each value is the mean tumor volume ± SD. *P values. (**F**) Histological images of T24/Mock and T24/GRIK2 cells. H&E and GRIK2 immunohistochemical staining of T24/Mock and T24/GRIK2 tumors. Black bar is 50 μm.

GRIK2 was preferentially expressed in ALDH1^high^ cells derived from UM-UC3 cells. Expression of GRIK2 was also detectable in J82 cells, but it was not detected in T24 cells (Figure 4A). To elucidate the functions of GRIK2 we established *GRIK2* overexpressed cells of UM-UM3 cells and T24 cells. *GRIK2* gene expression and GRIK2 protein expression were confirmed by RT-PCR and immunohistochemical staining (Figure 4B and 4F). Matrigel invasion assay, sphere-forming assay and xenograft transplantation in NOD/SCID mice using *GRIK2* stable transformants were performed. The matrigel invasion assay revealed that overexpression of GRIK2 increased the invasion ability of T24 cells (*P* < 0.05) (Figure 4C). *GRIK2*-overexpressed cells showed greater sphere-forming ability than that of control plasmid transfected (T24/Mock) cells (*P* < 0.05) (Figure 4D). A limiting dilution assay revealed that overexpression of GRIK2 significantly increased the frequencies of CSCs/CICs of UM-UC3 cells and T24 cells (Table 1). To evaluate tumorigenicity ability *in vivo*, xenograft transplantation of T24/GRIK2 and T24/Mock cells in NOD/SCID mice was performed. T24/GRIK2 cells initiated the formation of tumors in all 3 mice by day 63, whereas T24/Mock cells initiated the formation of tumors in 2 of the 3 mice by day 63. Tumors derived from T24/GRIK2 cells were significantly larger than those derived from T24/Mock cells (*P* < 0.05) (Figure 4E).

### GRIK2 and ALDH1 expression and clinicopathological features in patients with upper urinary tract urothelial carcinoma

**Figure 5:**
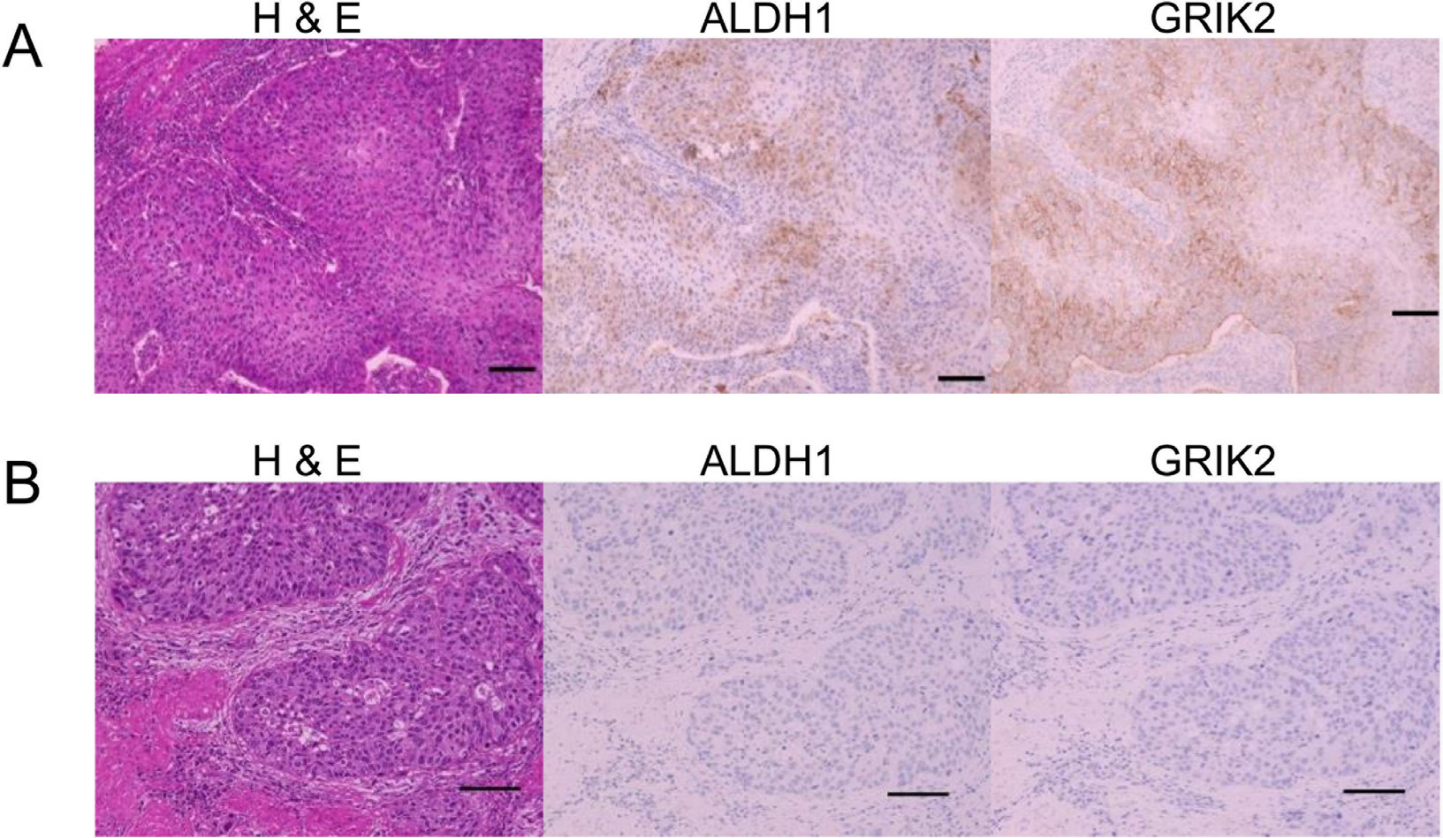
Histological images of H&E, ALDH1 immunohistochemical staining and GRIK2 immunohistochemical staining (**A**) Positive ALDH1 and GRIK2 expression in tumor cells. (**B**) Negative ALDH1 and GRIK2 expression in tumor cells.

We previously described ALDH1 expression is associated with poor prognosis in patients with upper urinary tract UC [15], and ALDH1 is a well accepted marker for CSCs/CICs. However, direct relation between ALDH1 and CSCs/CICs is still elusive. To elucidate ALDH1 in CSCs/CICs, we established *ALDH1A1* overexpressed cells using ALDH1-negative cells (5637 cells and T24 cells) (Supplementary Figure 4). Limiting dilution assay revealed that overexpression of ALDH1A1 in 5637 cells significantly increased CSCs/CICs frequency, and T24 cells *ALDH1A1* overexpressed cells showed tendency of increase CSCs/CICs frequency but the difference did not reach statistical significance (Table 1). Thus, both GRIK2 and ALDH1 are related to CSCs/CICs; however, ALDH1A1 did not change the expression of GRIK2 in 5637 cells (Supplementary Figure 4). To evaluate the relationship between ALDH1 and GRIK2, we analyzed data for 113 patients who underwent radical nephroureterectomy (Table 2). GRIK2-positive cells and ALDH1-positive cells (Figure 5A) were observed in 87 (77%) and 36 (32%) of the patients, respectively. GRIK2-negative cells and ALDH1-negative cells were observed in 26 (23%) and 77 (68%) of the patients (Figure 5B), respectively. GRIK2-positive cases showed a higher rate of lymph node metastasis (13%, *P* = 0.047), high grade of tumor (86%, *P* = 0.0002) and lymphovascular invasion (37%, *P* = 0.009) compared with GRIK2-negative cases. ALDH1-positive cases showed a higher rate of pathological T stage (pT2 or more: 77%, *P* = 0.020), high grade of tumor (92%, *P* = 0.0099) and lymphovascular invasion (47%, *P* = 0.012) (Table 3). These results indicated that GRIK2 and ALDH1 were associated with high invasion ability.

**Table 2:** Characteristics of the 113 patients

Characteristics	
Median age in years (range)	69 (32–88)
Median follow-up in months (range)	27 (2–142)
Sex	
Male	80 (71)
Female	33 (29)
Side	
Right	50 (44)
Left	62 (55)
Bilateral	1 (1)
Primary site (main)	
Renal pelvis	64 (57)
Ureter	44 (39)
Both	5 (4)
Pathological stage	
Stage 0a	15 (13)
Stage 0is	2 (2)
Stage I	15 (13)
Stage II	22 (20)
Stage III	44 (39)
Stage IV	15 (13)
Chemotherapy	
Neoadjuvant	8 (7)
Adjuvant	5 (4)

Values are n (%) except where mentioned otherwise.

**Table 3:** GRIK2/ALDH1 expression and pathological factors in patients with upper urinary tract urothelial carcinoma

Variable	GRIK2			ALDH1		
	Positive (%)	Negative (%)	*p*-value	Positive (%)	Negative (%)	*p*-value
Number of patients	87 (77)	26 (23)		36 (32)	77 (68)	
Pathological T stage			0.126			0.020
pTa	11 (13)	4 (15)		4 (11)	11 (14)	
pTis	1 (1)	1 (4)		2 (6)	0 (0)	
pT1	8 (9)	8 (31)		2 (6)	14 (18)	
pT2	20 (23)	4 (15)		9 (25)	15 (20)	
pT3	43 (49)	8 (31)		15 (41)	36 (47)	
pT4	4 (5)	1 (4)		4 (11)	1 (1)	
Pathological N stage			0.047			0.412
pN0	76 (87)	26 (100)		31 (86)	71 (92)	
pN1	5 (6)	0 (0)		3 (8)	2 (3)	
pN2	6 (7)	0 (0)		2 (6)	4 (5)	
Grade			0.0002			0.0099
Low	12 (14)	13 (50)		3 (8)	22 (29)	
High	75 (86)	13 (50)		33 (92)	55 (71)	
Lymphovascular invasion			0.009			0.012
Negative	55 (63)	23 (88)		19 (53)	59 (77)	
Positive	32 (37)	3 (12)		17 (47)	18 (23)	

**Figure 6:**
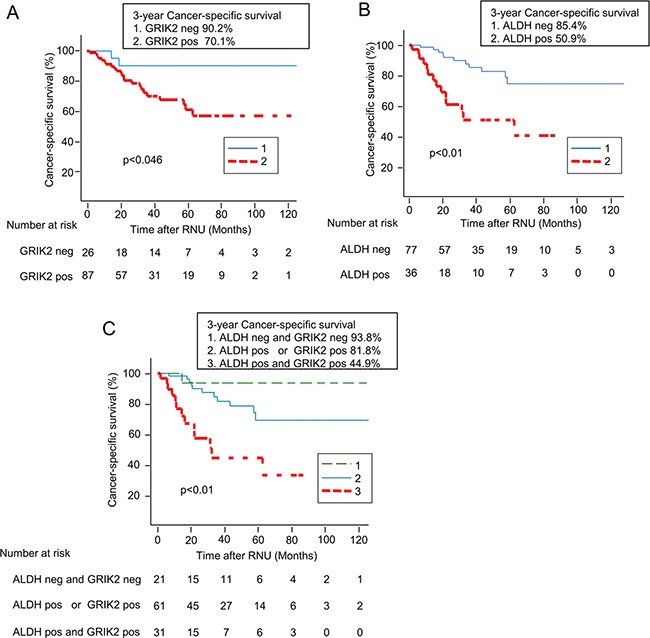
Kaplan-Meier curves for cancer-specific survival rates according to (**A**) GRIK2 expression status, (**B**) ALDH1 expression status and (**C**) combined expression status of GRIK2 and ALDH1.

We evaluated the association of GRIK2 and/or ALDH1 with survival and recurrence. The 3-year cancer-specific survival rates of patients with GRIK2-positive and GRIK2–negative tumors were 90.2% and 70.1% (*P* = 0.046), respectively (Figure 6A). The 3-year cancer-specific survival rates of patients with ALDH1-positive and ALDH1-negative tumors were 85.4% and 50.9% (*P* < 0.01), respectively (Figure 6B). Thus, both the expression of GRIK2 and that of ALDH1 were correlated with poorer prognosis. To evaluate the impact of the combination of GRIK2 and ALDH1, we classified the patients into three groups according to the immunohistochemical staining of GRIK2 and ALDH1: category 1 (both GRIK2 and ALDH1 negative), category 2 (GRIK2 or ALDH1 positive), and category 3 (both GRIK2 and ALDH1 positive) (Supplementary Table 2). The 3-year cancer-specific survival rates of patients in categories 1, 2 and 3 were 93.8%, 81.8% and 44.9%, respectively (*P* < 0.01, Figure 6C). However, intravesical recurrence-free survival rates of patients with GRIK2 and/or ALDH1 expressing tumors were not significantly different (*P* = 0.279, Supplementary Figure 5).

These results indicated that the novel UC CSC/CIC-related gene GRIK2 and ALDH1A1 have a role in the maintenance of UC CSCs/CICs and that the expression of GRIK2 combined with ALDH1 is a novel prognostic marker for UC cases.

## DISCUSSION

In this study, we successfully isolated urothelial carcinoma CSCs/CICs using the UC cell line UM-UC3 based on the ALDH1 activity assay and identified the glutamate receptor, ionotropic, kainate 2 (GRIK2) gene as a gene involved in the invasion ability of ALDH1 high activity cells. To our knowledge, this is the first report showing that ALDH1^high^ cells can also be isolated from UM-UC3 urothelial carcinoma cells by the ALDEFLUOR assay and that ALDH1^high^ cells have stem cell characteristics.

The UC cell lines RT4, T24 and 5637 or TCCSUP, T24 and 5637 were used in previous studies on ALDH1 activity of UC CSCs/CICs [8, 21]. However, RT-PCR and Western blot showed that *ALDH1A1* and ALDH1 were not expressed in T24 and 5637 cells, in this study. RT4 cells were positive in the ALDEFLUOR assay, but ALDH1^high^ cells derived from RT4 cells did not show grater sphere-forming ability than that of ALDH1^low^ cells. Thus, we did not use RT4 cells for xenograft transplantation in NOD/SCID mice. However, in a previous study, ALDH1^high^ cells derived from RT4 cells showed high colony-forming ability by a soft agar assay and high tumorigenicity ability by xenograft transplantation in NOD/SCID mice [8]. These different results might be due to differences in assay and cell culture conditions. TCCSUP cells showed a high level of ALDH1 expression, but ALDH1^high^ cells derived from TCCSUP cells did not show high sphere-forming ability, and these results are compatible with results of a previous study. [21] ALDH1 was expressed in TCCSUP and J82 cells; however, only a few spheres were observed in the sphere-forming assay. This result might have been caused by mechanical damage caused by the cell sorter because only a few colonies were observed in the condition with 10% FBS. Thus, UM-UC3 is a reasonable source for study in UC CSCs/CICs.

GRIK2 is one of the glutamate receptors that is the predominant neurotransmitter receptor in the mammalian brain and is activated in various normal neurophysiologic processes. Glutamate receptors are membrane proteins and are classified into two major groups: metabotropic glutamate receptors and ionotropic glutamate receptors. The ionotropic glutamate receptors include *N*-methyl-D-asparate (NMDA), α-amino-3-hydroxy-5-methyliso-xazole-4-propionate (AMPA) and kainate glutamate receptors. GRIK2 (or GluR6) is one of the five members of the kainate glutamate receptor subgroup, which also includes GluR5-7, KA1 and KA2. Although, kainate receptors are distributed throughout the central nervous system, their physiological significance in cancer cells is not known well [17, 20, 22].

There have been a few reports in which the association between GRIK2 and cancers was described. GRIK2 was reported to be a potential tumor suppressor gene in gastric cancer [17]. GRIK2 was expressed in normal gastric tissue; however, its expression was repressed in gastric cancer tissues by GRIK2 promoter region methylation. Furthermore, overexpression of GRIK2 in gastric cancer cells decreased colony formation and cell migration. These findings indicate that *GRIK2* is a tumor suppressor gene. Recently, polymorohism of GRIK2 TT (rs1335022) was associated with oral cancer [19]. However, the SNP region is in an intron and the SNP does not alter mature GRIK2 protein sequence. Our findings indicate oncogenic potential of GRIK2 in UCs. Expression of GRIK2 was not seen in the normal urothelial epithelium, and GRIK2 expression was found in UC tissues. Functional assays revealed that GRIK2 has roles in invasion ability, sphere-forming ability and tumorigenicity *in vivo*. Furthermore, GRIK2 expression in UC tissues was associated with pathological N stage, tumor grade and lymphovascular invasion (Table 2). Molecular mechanisms of GRIK2 is still elusive in current study; however, *GRIK2* knockdown experiments revealed that GRIK2 expression is related to expression of ALDH1A1 (data not shown) and ALDH^high^ cells by ALDEFLUOR assay. Thus, GRIK2 might have a role in ALDH^high^ cells. These results indicated that GRIK2 has different roles in gastric cancer and urothelial cancer.

Upper urinary tract urothelial carcinomas including renal pelvic caner and ureteral cancer are uncommon and account for only 5–10% of UCs [23]. Patients with localized upper urinary tract carcinoma usually underwent total nephroureterctomy for standard therapy. It is challenging to predict recurrence, and upper urinary tract carcinoma patients with recurrence have a very poor prognosis, even if systemic chemotherapy is performed. Expression of GRIK2 and that of ALDH1 were associated with poor prognosis after nephroureterectomy. These results indicated that GRIK2 expression and ALDH1 expression in patients with UC could be prognostic factors after nephroureterectomy.

In summary, we found that a UC cell line with high ALDH1 activity had CSC/CIC properties. GRIK2 is expressed in UC CSCs/CICs and has roles in invasion, sphere formation and tumorigenicity. GRIK2 expression combined with ALDH1 activity might be a novel prognostic marker for UCs.

## MATERIALS AND METHODS

### Ethics statement

Mice were maintained and experimented on in accordance with the guidelines after approval by the Committee of Sapporo Medical University (No.10-032). All studies were approved by Institutional Review Boards (IRB) of Sapporo Medical University (No. 22–45).

### Cell lines

Human urothelial carcinoma cell lines UM-UC3, T24, HT1376, SW780, TCCSUP, 5637 and J82 (American Type Culture Collection (ATCC), Manassas, VA, USA) were cultured in Dulbecco's modified Eagle's medium (DMEM) (Sigma-Aldrich, St Louis, MO, USA), and the human urothelial papilloma cell line RT4 (ATCC) was cultured in McCoy's 5A medium (Life Technologies, Carlsbad, CA, USA) supplemented with 10% fetal bovine serum (FBS). PLAT-A cells (kind gifts from Dr. T Kitamura, Tokyo, Japan) were cultured in DMEM supplemented with 10% FBS, 10 μg/ml blasticidin and 1 μg/ml puromycin (Sigma-Aldrich). Cells were incubated in a 37°C incubator with humidified air and 5% CO_2_.

### ALDEFLUOR assay and fluorescence-activated cell sorting isolation

Aldehyde dehydrogenase 1 (ALDH1) activity was measured as described previously using the ALDEFLUOR assay kit (STEMCELL Technologies, Vancouver, Canada) [9]. Briefly, cells were suspended in ALDEFLUOR assay buffer containing the ALDH1 substrate boron-dipyrromethene-aminoacetaldehyde (1 μmol/l/1 × 10^6^ cells) and incubated for 30 minutes at 37°C. As a negative control, aliquots of each sample were treated with 50 mmol/l of the specific ALDH1 inhibitor diethylaminobenzaldehyde. Stained cells were analyzed using a FACSAria II cell sorter. Sorting gates were established using propidium iodide-stained cells for viability.

### RNA preparation and RT-PCR analysis

Total RNA isolation and RT-PCR analysis were performed as described previously [24]. Human Multiple Tissue cDNA Panels I and II (Takara Bio Inc., Otsu, Japan) were used as templates of normal adult tissue cDNAs. The primer pairs used for RT-PCR analysis were 5′-TGTTAGCTGATGCCGACTTG-3′ and 5′-CATCACAACCACGTTTCCAG-3′ for *ALDH1A1* with an expected PCR product size of 154 bp, 5′- TACCCAGCGGAACTGTAACC-3′ and 5′- CCTCCACCATTTCTCCTTCA-3′ for *GRIK2* with an expected PCR product size of 166 bp, and 5′- ACCACAGTCCATGCCATCAC-3′ and 5′- TCCACCACCCTGTTGCTGTA-3′ for *GAPDH* with an expected PCR product size of 452 bp.

### Western blot

Western blots were performed as described previously [25]. Briefly, 1 × 10^5^ of ALDH1^high^ cells and ALDH1^low^ cells were lysed in 40 μL of SDS sample buffer. Anti-ALDH1 mouse monoclonal antibody (clone: 44/ALDH; BD Biosciences, San Jose, CA, USA) was used at 500-times dilution. Anti-β-actin and anti-α-tubulin mouse monoclonal antibodies (Sigma-Aldrich) were used as loading controls at 2000-times dilution. Anti-mouse IgG and anti-rabbit IgG second antibodies (KPL, Gaithersburg, MD, USA) were used at 2000-times dilution. The membrane was visualized with Chemiluminescent HRP Substrate (Millipore, Billerica, MA, USA) according to the manufacturer's protocol, and pictures were taken by an Odyssey Fc imaging System (LI-COR, Lincoln, NE, USA).

### Sphere-forming assay

A total of 2 × 10^2^ sorted ALDH1^high^ and ALDH1^low^ cells derived from UM-UC3, TCCSUP, J82 and RT4 cells, 5 × 10^2^ si-mediated *GRIK2* knockdown cells or T24/Mock and T24/GRIK2 cells were incubated in DMEM/F12 medium (Life Technologies) supplemented with 10 ng/ml basic fibroblast growth factor and 20 ng/ml epidermal growth factor (Sigma-Aldrich) in each well of an Ultra-Low Attachment Surface culture 6-well plate (Sigma-Aldrich). T24/Mock and T24/GRIK2 cells were incubated with an additional 0.5 μg/ml insulin (Sigma-Aldrich). Spheres larger than 100 μm were counted at day 4 or day 5 by light microscopy.

For estimation of CSC/CIC frequency, limiting dilution analysis was performed. Serially diluted cells (1/well–1000/well) were cultured in 96-well Ultra-Low Attachment plate and cultured for 7–10 days. Then sphere-positive wells were counted. The estimation of CSC/CIC frequency was calculated by ELDA web site (http://bioinf.wehi.edu.au/software/elda/) [26].

### Xenograft transplantation in NOD/SCID mice

Isolated ALDH1^high^ and ALDH1^low^ cells or T24/Mock and T24/GRIK2 cells were resuspended at concentrations of 1 × 10^3^ cells in phosphate buffered saline and Matrigel (BD Biosciences) mixture (1:1). ALDH1^high^ and ALDH1^low^ cells were injected subcutaneously into the right and left mid back areas of anesthetized non-obese diabetic/severe combined immunodeficient (NOD/SCID) female mice (Charles River Laboratory Japan, Yokohama, Japan) at the age of 4–6 weeks. Tumor growth was monitored weekly, and tumor volume was calculated by XY^2^/2 (X = long axis, Y = short axis).

### Matrigel invasion assay

Cellular potential for invasiveness was determined using Matrigel invasion chambers (BD Biosciences) according to the manufacturer's instructions [10]. Briefly, a total of 1 × 10^4^ sorted ALDH1^high^ and ALDH1^low^ cells, 2 × 10^4^ si-mediated *GRIK2* knockdown cells or T24/Mock and T24/GRIK2 cells were seeded in the upper chambers in serum-free DMEM. The outer chambers were filled with the same medium but with 10% FBS as a chemoattractant. Cells were incubated for 24 to 48 hours. Invasive cells were stained with hematoxylin, mounted on slides, and counted by light microscopy. Each experiment was repeated 3 times.

### Resistance to cisplatin

Isolated ALDH1^high^ and ALDH1^low^ cells were seeded in 96-well plates at 2×10^3^ cells per well. The cells in both populations were treated with cisplatin (0.5–5.0 μg/ml). After 48 hours of incubation, viability of the cells was determined using the cell counting kit WST-1 (Dojindo Molecular Technologies, Kumamoto, Japan).

### cDNA microarray

RNA from ALDH1^high^ cells was labeled with Cy5 dye, and RNA from ALDH1^low^ cells was labeled with Cy3 dye. The probe mixture was hybridized for 40 hours at 65°C to a G4412A Human Genome CGH Microarray Kit 105A (Agilent Technologies, Tokyo, Japan). The array was scanned after washing with a G2565BA Microarray Scanner, and fluorescent signals were acquired using Feature Extraction software (Agilent Technologies). The average expression ratio of Cy5 to Cy3 was determined per gene.

A dye swap experiment was also done to label ALDH1^high^ and ALDH1^low^ cells with Cy3 and Cy5, respectively. An average ratio of more than 2.0, reproducible in two experiments, was determined to indicate differential up-regulation in ALDH1^high^ cells [10]. Microarray raw data and processed data have been deposited in the NCBI GEO database (GSE66684).

### siRNA-mediated knockdown

GRIK2 siRNAs (SR301950A, SR301950B and SR301950B) were purchased from OriGene Technologies (Rockville, MD, USA). siRNA transfection was performed using lipofectamine RNAi MAX reagent (Life Technologies) according to the protocol of the manufacturer. Cells were transfected with siRNA at final concentration of 25 pmol/ 2 mL, 24 hours before analysis. Non-targeting siRNA (OriGene) was used as a negative control. *GRIK2* knockdown was confirmed by quantitative real-time PCR.

### Retroviral gene transduction and generation of stable transformants

Transduction of genes into cells was performed by a retrovirus-mediated method as described previously [27]. PLAT-A cells were transiently transduced with a pMXs-puro (kind gift from Dr. T. Kitamura, Tokyo, JAPAN) retroviral vector expressing GRIK2 and a control plasmid using FuGENE HD transfection reagent (Promega, Tokyo, Japan) following the manufacturer's protocol. Retroviral supernatants were harvested 48 hours after transfection. The supernatant was used for infection of T24 cells in the presence of 8 μg/ml of polybrene (Sigma-Aldrich) overnight. For the generation of stable transformants, the infected cells were selected with 1 μg/ml puromycin (Sigma-Aldrich).

### Quantitative real-time PCR analysis

Quantitative real-time PCR was performed as previously described. [28] *ALDH1A1*, *GRIK2* and *GAPDH* probes were designed by the manufacturer (TaqMan Gene expression assay; Life Technologies).

### Immunohistochemical staining and scoring

Immunohistochemical (IHC) staining and scoring were done as previously described [15]. To detect ALDH1 and GRIK2 proteins, we used anti-ALDH1 mouse monoclonal antibody (clone: 44/ALDH; BD Biosciences) at 1000-times dilution and anti-GRIK2 rabbit monoclonal antibody (clone: EPR6307; abcam, Tokyo, Japan) at 500-times dilution. All specimens were reviewed by a pathologist who was blinded to clinicopathological data (YH). For ALDH1, a tumor presenting at least one ALDH1-positive cancer cell was considered to be ALDH1-positive. For GRIK2, a tumor presenting more than 1% GRIK2-positive cancer cells was considered to be GRIK2-positive.

### Patients

We reviewed the clinical pathology archives of 113 patients who underwent radical nephroureterectomy because of upper urinary tract urothelial carcinoma at Sapporo Medical University Hospital from January 1998 to September 2011.

### Statistical analysis

Data are presented as means ± SD. Differences in variables were assessed using Student's *t-test*. Survival curves were constructed according to the Kaplan-Meier method. Statistical significance was determined by the log-rank test. *P* < 0.05 was considered significant. Statistical analysis was done with jmp9 software (SAS institute).

## SUPPLEMENTARY MATERIALS FIGURES AND TABLES





## Abbreviations

CSC/CIC: cancer stem-like cell/cancer-initiating cell, CIC
UC: urothelial carcinoma
ALDH1: aldehyde dehydrogenase 1A1
GRIK2: glutamate receptor, ionotropic, kainate 2
DEAB: diethylaminobenzaldehyde.
