# Supplementary material for: GRIK2 has a role in the maintenance of urothelial carcinoma stem-like cells, and its expression is associated with poorer prognosis
Source: Oncotarget. 2017 Mar 16;8(17):28826–39. doi: 10.18632/oncotarget.16259 (PMC5438695; doi:10.18632/oncotarget.16259)
Supplement: Supplementary file 1 [file oncotarget-08-28826-s001.pdf]

GRIK2 has a role in the maintenance of urothelial carcinoma stem-like cells, and its expression is associated with poorer prognosis

Supplementary Materials

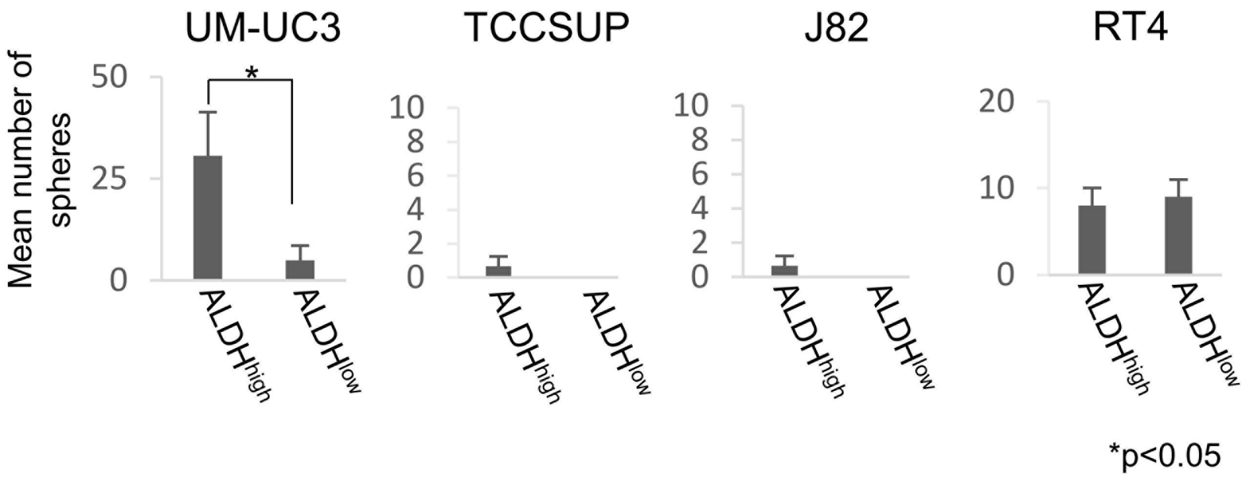

Supplementary Figure 1: Sphere-forming assay. ALDH1high and ALDH1Low cells derived from UM-UC3, TCCSUP, J82 and RT4 were incubated in Dulbecco's modified eagles medium (DMEM)/F12 media with 10% FBS. Each values is the mean number of spheres ± SD **P* values.

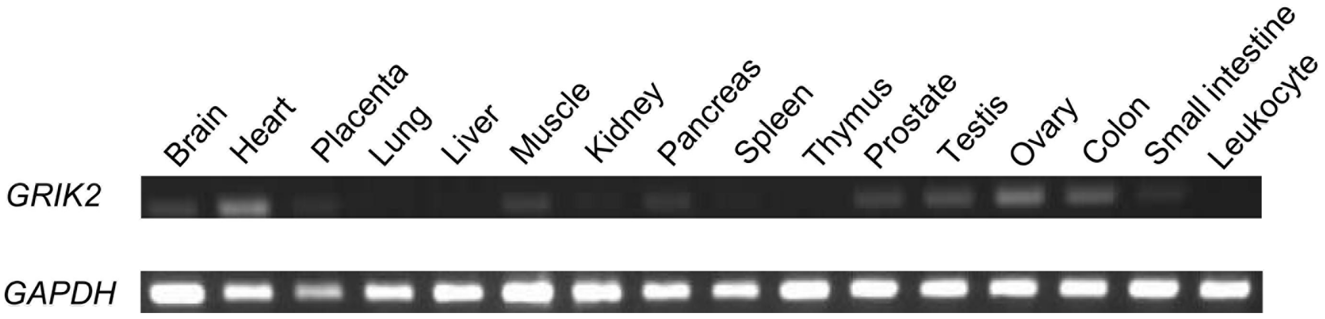

Supplementary Figure 2: RT-PCR analysis with normal organs. Expression of *GRIK2* mRNA was examined by RT-PCR. GAPDH was used as a positive control.

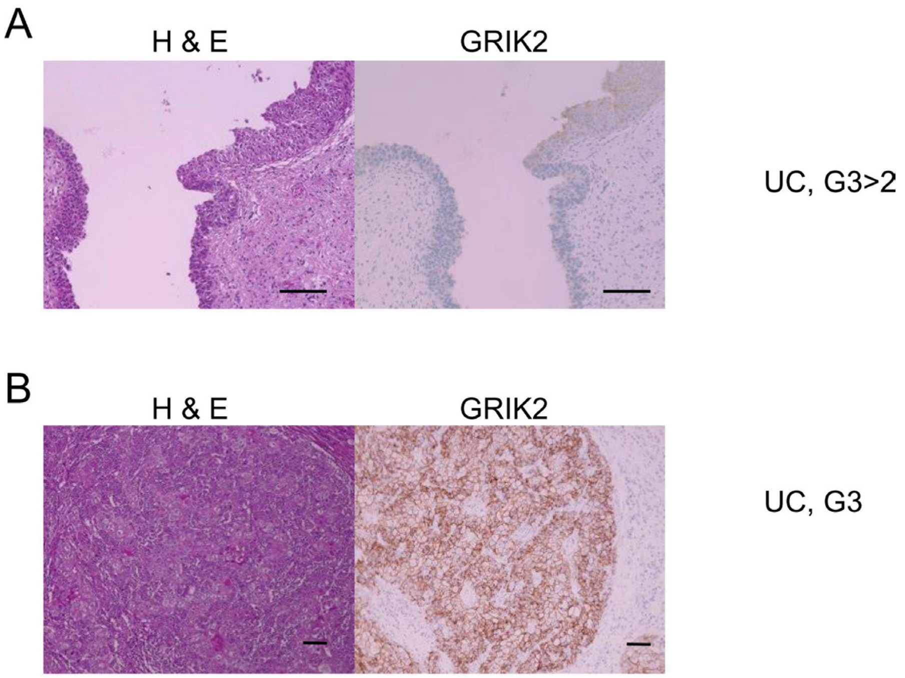

Supplementary Figure 3: Histological images of urothelial carcinoma and normal organs. (**A** and **B**) Hematoxylin-Eosin Staining (H&E) and GRIK2 immunohistochemical staining of urothelial carcinoma. Black bar is 100 µm. Black bar is 100 µm.

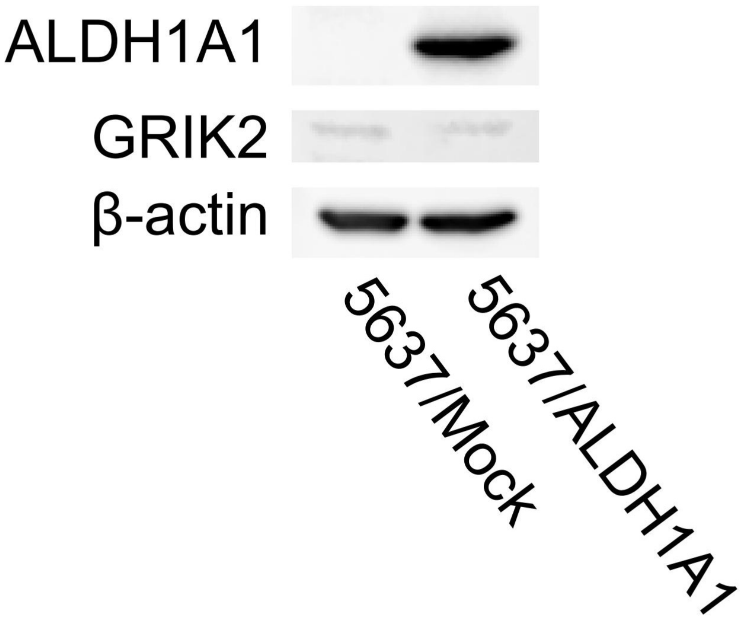

Supplementary Figure 4: Establishment of ALDH1A1 overexpressed 5637 cells. ALDH1A1 overexpressed 5637 cells were analyzed by an Western blot. β-actin was used as an internal positive control.

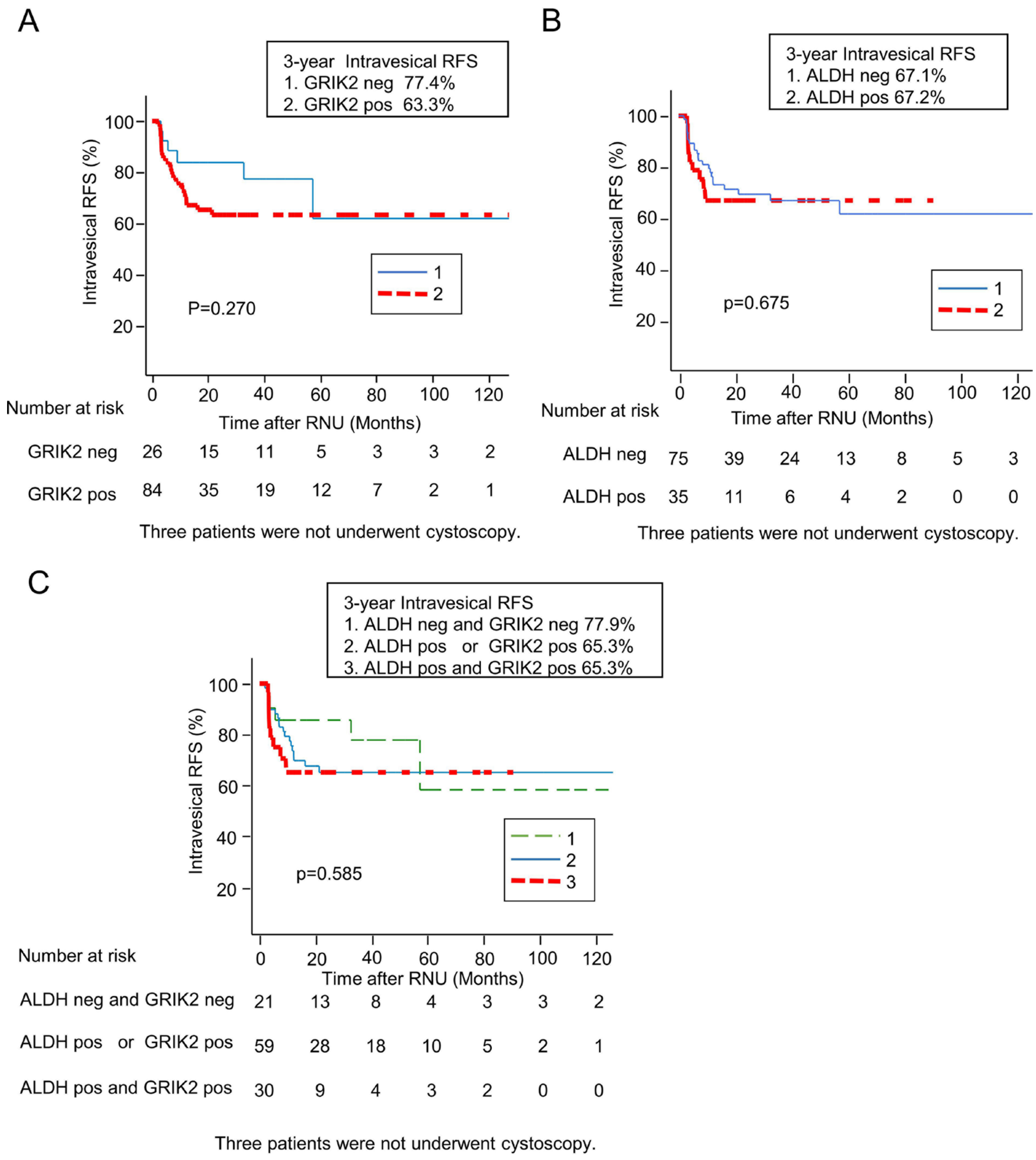

Supplementary Figure 5: Kaplan-Meier curves for intravesical recurrence-free survival rates according to (**A**) GRIK2 expression status, (**B**) ALDH1 expression status and (**C**) combined expression status of GRIK2 and ALDH1.

Supplementary Table 1: Summary of genes overexpressed in ALDH^{high} cells. See Supplementary_Table_1

Supplementary Table 2: Frequency of positive expression of GRIK2 and ALDH1

Category	Markers	*n* (%)
1	GRIK2 pos ALDH1 pos	31 (27)
2	GRIK2 pos ALDH1 neg	56 (50)
	GRIK2 neg ALDH1 pos	5 (4)
3	GRIK2 neg ALDH1 neg	21 (19)

pos: positive, neg: negative.
