# Supplementary material for: GRIK2 has a role in the maintenance of urothelial carcinoma stem-like cells, and its expression is associated with poorer prognosis
Source: Oncotarget. 2017 Mar 16;8(17):28826–39. doi: 10.18632/oncotarget.16259 (PMC5438695; doi:10.18632/oncotarget.16259)
Supplement: Supplementary file 2 [file oncotarget-08-28826-s002.doc]

| Table S1. Summary of genes overexpressed in ALDH^high^ cells | | | | |
| --- | --- | --- | --- | --- |
| GeneName | SystematicName | Description | Cy5/Cy3 | Cy3/Cy5 |
| SAMSN1 | NM_022136 | ref|Homo sapiens SAM domain, SH3 domain and nuclear localization signals 1 (SAMSN1), mRNA [NM_022136] | 22.91 | 8.82 |
| ALDH1A1 | NM_000689 | ref|Homo sapiens aldehyde dehydrogenase 1 family, member A1 (ALDH1A1), mRNA [NM_000689] | 18.56 | 14.61 |
| INHBA | NM_002192 | ref|Homo sapiens inhibin, beta A (INHBA), mRNA [NM_002192] | 9.94 | 2.23 |
| C7orf58 | NM_024913 | ref|Homo sapiens chromosome 7 open reading frame 58 (C7orf58), transcript variant 1, mRNA [NM_024913] | 9.36 | 4.93 |
| INHBA | NM_002192 | ref|Homo sapiens inhibin, beta A (INHBA), mRNA [NM_002192] | 4.75 | 3.12 |
| INHBA | NM_002192 | ref|Homo sapiens inhibin, beta A (INHBA), mRNA [NM_002192] | 4.65 | 2.61 |
| ALDH1A1 | NM_000689 | ref|Homo sapiens aldehyde dehydrogenase 1 family, member A1 (ALDH1A1), mRNA [NM_000689] | 4.12 | 7.28 |
| ENST00000494145 | ENST00000494145 | gb|Homo sapiens cDNA, FLJ17074. [AK310032] | 3.96 | 2.51 |
| LRRC6 | NM_012472 | ref|Homo sapiens leucine rich repeat containing 6 (LRRC6), mRNA [NM_012472] | 3.82 | 2.74 |
| LRRC6 | NM_012472 | ref|Homo sapiens leucine rich repeat containing 6 (LRRC6), mRNA [NM_012472] | 3.69 | 2.49 |
| LRRC6 | NM_012472 | ref|Homo sapiens leucine rich repeat containing 6 (LRRC6), mRNA [NM_012472] | 3.66 | 3.05 |
| ENST00000409758 | ENST00000409758 | Unknown | 3.65 | 3.88 |
| C7orf58 | NM_024913 | ref|Homo sapiens chromosome 7 open reading frame 58 (C7orf58), transcript variant 1, mRNA [NM_024913] | 3.63 | 2.76 |
| INHBA | NM_002192 | ref|Homo sapiens inhibin, beta A (INHBA), mRNA [NM_002192] | 3.35 | 2.69 |
| INHBA | NM_002192 | ref|Homo sapiens inhibin, beta A (INHBA), mRNA [NM_002192] | 3.34 | 2.54 |
| INHBA | NM_002192 | ref|Homo sapiens inhibin, beta A (INHBA), mRNA [NM_002192] | 3.33 | 2.51 |
| LRRC6 | NM_012472 | ref|Homo sapiens leucine rich repeat containing 6 (LRRC6), mRNA [NM_012472] | 3.33 | 2.02 |
| LOC100507949 | XR_111437 | ref|PREDICTED: Homo sapiens hypothetical LOC100507949, transcript variant 1 (LOC100507949), partial miscRNA [XR_111437] | 3.19 | 2.42 |
| ENST00000420762 | ENST00000420762 | gb|601883092F1 NIH_MGC_57 Homo sapiens cDNA clone IMAGE:4095431 5', mRNA sequence [BF216856] | 3.19 | 2.00 |
| LRRC6 | NM_012472 | ref|Homo sapiens leucine rich repeat containing 6 (LRRC6), mRNA [NM_012472] | 3.18 | 2.42 |
| ENST00000372550 | ENST00000372550 | ens|peptidylprolyl isomerase H (cyclophilin H) [Source:HGNC Symbol;Acc:14651] [ENST00000372550] | 3.15 | 7.54 |
| HDC | NM_002112 | ref|Homo sapiens histidine decarboxylase (HDC), mRNA [NM_002112] | 3.14 | 5.34 |
| FLRT3 | NM_198391 | ref|Homo sapiens fibronectin leucine rich transmembrane protein 3 (FLRT3), transcript variant 2, mRNA [NM_198391] | 3.11 | 2.91 |
| INHBA | NM_002192 | ref|Homo sapiens inhibin, beta A (INHBA), mRNA [NM_002192] | 3.09 | 2.66 |
| SEMA3C | NM_006379 | ref|Homo sapiens sema domain, immunoglobulin domain (Ig), short basic domain, secreted, (semaphorin) 3C (SEMA3C), mRNA [NM_006379] | 3.08 | 2.23 |
| IRX4 | NM_016358 | ref|Homo sapiens iroquois homeobox 4 (IRX4), mRNA [NM_016358] | 3.07 | 2.23 |
| LRRC6 | NM_012472 | ref|Homo sapiens leucine rich repeat containing 6 (LRRC6), mRNA [NM_012472] | 2.98 | 2.50 |
| INHBA | NM_002192 | ref|Homo sapiens inhibin, beta A (INHBA), mRNA [NM_002192] | 2.93 | 2.97 |
| INHBA | NM_002192 | ref|Homo sapiens inhibin, beta A (INHBA), mRNA [NM_002192] | 2.90 | 2.81 |
| PID1 | NM_017933 | ref|Homo sapiens phosphotyrosine interaction domain containing 1 (PID1), transcript variant 1, mRNA [NM_017933] | 2.87 | 2.15 |
| EDA2R | NM_001199687 | ref|Homo sapiens ectodysplasin A2 receptor (EDA2R), transcript variant 1, mRNA [NM_001199687] | 2.83 | 2.62 |
| GRIK2 | NM_021956 | ref|Homo sapiens glutamate receptor, ionotropic, kainate 2 (GRIK2), transcript variant 1, mRNA [NM_021956] | 2.74 | 2.16 |
| SPTLC3 | NM_018327 | ref|Homo sapiens serine palmitoyltransferase, long chain base subunit 3 (SPTLC3), mRNA [NM_018327] | 2.73 | 2.06 |
| LRRC6 | NM_012472 | ref|Homo sapiens leucine rich repeat containing 6 (LRRC6), mRNA [NM_012472] | 2.71 | 2.51 |
| ENST00000506335 | ENST00000506335 | Unknown | 2.71 | 3.38 |
| PARP3 | NM_005485 | ref|Homo sapiens poly (ADP-ribose) polymerase family, member 3 (PARP3), transcript variant 2, mRNA [NM_005485] | 2.60 | 2.42 |
| NEIL3 | NM_018248 | ref|Homo sapiens nei endonuclease VIII-like 3 (E. coli) (NEIL3), mRNA [NM_018248] | 2.58 | 2.18 |
| NCRNA00281 | NR_027278 | ref|Homo sapiens non-protein coding RNA 281 (NCRNA00281), non-coding RNA [NR_027278] | 2.54 | 6.59 |
| MOBKL2B | NM_024761 | ref|Homo sapiens MOB1, Mps One Binder kinase activator-like 2B (yeast) (MOBKL2B), mRNA [NM_024761] | 2.49 | 2.78 |
| LARP1B | NM_018078 | ref|Homo sapiens La ribonucleoprotein domain family, member 1B (LARP1B), transcript variant 1, mRNA [NM_018078] | 2.47 | 3.95 |
| TLE4 | NM_007005 | ref|Homo sapiens transducin-like enhancer of split 4 (E(sp1) homolog, Drosophila) (TLE4), mRNA [NM_007005] | 2.43 | 2.07 |
| C1orf74 | NM_152485 | ref|Homo sapiens chromosome 1 open reading frame 74 (C1orf74), mRNA [NM_152485] | 2.40 | 2.02 |
| FBXO5 | NM_001142522 | ref|Homo sapiens F-box protein 5 (FBXO5), transcript variant 2, mRNA [NM_001142522] | 2.37 | 2.48 |
| LRRC6 | NM_012472 | ref|Homo sapiens leucine rich repeat containing 6 (LRRC6), mRNA [NM_012472] | 2.37 | 2.76 |
| PMFBP1 | NM_031293 | ref|Homo sapiens polyamine modulated factor 1 binding protein 1 (PMFBP1), transcript variant 1, mRNA [NM_031293] | 2.34 | 2.00 |
| AR | NM_000044 | ref|Homo sapiens androgen receptor (AR), transcript variant 1, mRNA [NM_000044] | 2.31 | 2.66 |
| RAB39B | NM_171998 | ref|Homo sapiens RAB39B, member RAS oncogene family (RAB39B), mRNA [NM_171998] | 2.29 | 3.45 |
| BCO2 | NM_031938 | ref|Homo sapiens beta-carotene oxygenase 2 (BCO2), transcript variant 1, mRNA [NM_031938] | 2.27 | 2.47 |
| CDK1 | NM_001786 | ref|Homo sapiens cyclin-dependent kinase 1 (CDK1), transcript variant 1, mRNA [NM_001786] | 2.27 | 2.70 |
| CDK1 | NM_001786 | ref|Homo sapiens cyclin-dependent kinase 1 (CDK1), transcript variant 1, mRNA [NM_001786] | 2.26 | 2.76 |
| CDK1 | NM_001786 | ref|Homo sapiens cyclin-dependent kinase 1 (CDK1), transcript variant 1, mRNA [NM_001786] | 2.26 | 2.71 |
| CDK1 | NM_001786 | ref|Homo sapiens cyclin-dependent kinase 1 (CDK1), transcript variant 1, mRNA [NM_001786] | 2.25 | 2.73 |
| CDK1 | NM_001786 | ref|Homo sapiens cyclin-dependent kinase 1 (CDK1), transcript variant 1, mRNA [NM_001786] | 2.24 | 2.74 |
| CDK1 | NM_001786 | ref|Homo sapiens cyclin-dependent kinase 1 (CDK1), transcript variant 1, mRNA [NM_001786] | 2.24 | 2.71 |
| CDK1 | NM_001786 | ref|Homo sapiens cyclin-dependent kinase 1 (CDK1), transcript variant 1, mRNA [NM_001786] | 2.24 | 2.75 |
| IRX4 | NM_016358 | ref|Homo sapiens iroquois homeobox 4 (IRX4), mRNA [NM_016358] | 2.22 | 2.32 |
| AK022341 | AK022341 | gb|Homo sapiens cDNA FLJ12279 fis, clone MAMMA1001743, weakly similar to Y BOX BINDING PROTEIN-1. [AK022341] | 2.22 | 2.21 |
| CDK1 | NM_001786 | ref|Homo sapiens cyclin-dependent kinase 1 (CDK1), transcript variant 1, mRNA [NM_001786] | 2.22 | 2.76 |
| CDK1 | NM_001786 | ref|Homo sapiens cyclin-dependent kinase 1 (CDK1), transcript variant 1, mRNA [NM_001786] | 2.21 | 2.69 |
| AR | NM_000044 | ref|Homo sapiens androgen receptor (AR), transcript variant 1, mRNA [NM_000044] | 2.20 | 2.53 |
| SLAIN2 | NM_020846 | ref|Homo sapiens SLAIN motif family, member 2 (SLAIN2), mRNA [NM_020846] | 2.20 | 3.15 |
| PCDH9 | NM_203487 | ref|Homo sapiens protocadherin 9 (PCDH9), transcript variant 1, mRNA [NM_203487] | 2.19 | 2.22 |
| CDK1 | NM_001786 | ref|Homo sapiens cyclin-dependent kinase 1 (CDK1), transcript variant 1, mRNA [NM_001786] | 2.18 | 2.70 |
| HIST1H4C | NM_003542 | ref|Homo sapiens histone cluster 1, H4c (HIST1H4C), mRNA [NM_003542] | 2.16 | 2.10 |
| ZNF331 | NM_018555 | ref|Homo sapiens zinc finger protein 331 (ZNF331), transcript variant 1, mRNA [NM_018555] | 2.15 | 2.35 |
| ENST00000333488 | ENST00000333488 | ens|palladin, cytoskeletal associated protein [Source:HGNC Symbol;Acc:17068] [ENST00000333488] | 2.14 | 3.27 |
| SPTLC3 | NM_018327 | ref|Homo sapiens serine palmitoyltransferase, long chain base subunit 3 (SPTLC3), mRNA [NM_018327] | 2.14 | 2.11 |
| ID4 | NM_001546 | ref|Homo sapiens inhibitor of DNA binding 4, dominant negative helix-loop-helix protein (ID4), mRNA [NM_001546] | 2.13 | 2.04 |
| ARL13B | NM_182896 | ref|Homo sapiens ADP-ribosylation factor-like 13B (ARL13B), transcript variant 1, mRNA [NM_182896] | 2.12 | 2.53 |
| NRG1 | NM_004495 | ref|Homo sapiens neuregulin 1 (NRG1), transcript variant HRG-gamma, mRNA [NM_004495] | 2.11 | 2.24 |
| BSDC1 | NM_001143888 | ref|Homo sapiens BSD domain containing 1 (BSDC1), transcript variant 1, mRNA [NM_001143888] | 2.11 | 2.11 |
| AR | NM_000044 | ref|Homo sapiens androgen receptor (AR), transcript variant 1, mRNA [NM_000044] | 2.11 | 2.58 |
| IRX4 | NM_016358 | ref|Homo sapiens iroquois homeobox 4 (IRX4), mRNA [NM_016358] | 2.09 | 3.48 |
| AR | NM_000044 | ref|Homo sapiens androgen receptor (AR), transcript variant 1, mRNA [NM_000044] | 2.06 | 2.55 |
| ENST00000358739 | ENST00000358739 | ens|histone cluster 1, H2ai [Source:HGNC Symbol;Acc:4725] [ENST00000358739] | 2.06 | 2.14 |
| NRG1 | NM_004495 | ref|Homo sapiens neuregulin 1 (NRG1), transcript variant HRG-gamma, mRNA [NM_004495] | 2.06 | 2.22 |
| ENST00000463718 | ENST00000463718 | gb|Homo sapiens brain organic anion transporter subtype OATP1b (OATP) mRNA, complete cds. [AF085224] | 2.05 | 2.08 |
| AR | NM_000044 | ref|Homo sapiens androgen receptor (AR), transcript variant 1, mRNA [NM_000044] | 2.05 | 2.61 |
| ENST00000370684 | ENST00000370684 | ens|protein kinase, cAMP-dependent, catalytic, beta [Source:HGNC Symbol;Acc:9381] [ENST00000370684] | 2.05 | 4.08 |
| AR | NM_000044 | ref|Homo sapiens androgen receptor (AR), transcript variant 1, mRNA [NM_000044] | 2.02 | 2.62 |
| ESCO2 | NM_001017420 | ref|Homo sapiens establishment of cohesion 1 homolog 2 (S. cerevisiae) (ESCO2), mRNA [NM_001017420] | 2.01 | 2.41 |
